# Quantitative Anti-HBc in Liver Pathological States in Patients with Chronic Hepatitis B Virus Infection

**DOI:** 10.1155/2019/6545642

**Published:** 2019-12-25

**Authors:** Zhan-qing Zhang, Bi-sheng Shi, Wei Lu, Dan-ping Liu, Dan Huang, Yan-ling Feng

**Affiliations:** ^1^Department of Hepatobiliary Medicine, Shanghai Public Health Clinical Center of Fudan University, Shanghai, China; ^2^Scientific Research Center, Shanghai Public Health Clinical Center of Fudan University, Shanghai, China; ^3^Department of Clinical Pathology, Shanghai Public Health Clinical Center of Fudan University, Shanghai, China

## Abstract

**Background:**

Changes of hepatitis B core antigen antibody (anti-HBc) in liver pathological involvement in patients with chronic hepatitis B virus (HBV) infection have not been investigated in detail. This study aimed to explore evolving patterns of anti-HBc following liver pathological states and to investigate validities of anti-HBc for predicting liver pathological states.

**Methods:**

254 HBeAg-positive and 237 HBeAg-negative patients with chronic HBV infection were enrolled. Liver pathological diagnoses referred to Scheuer standard, and anti-HBc was measured using chemiluminescence microparticle immunoassay.

**Results:**

Anti-HBc was significantly positively correlated with pathological grades and stages in both HBeAg-positive (*r*_*s*_ = 0.312, *P* < 0.0001, and *r*_*s*_ = 0.268, *P* < 0.0001) and HBeAg-negative (*r*_*s*_ = 0.270, *P* < 0.0001, and *r*_*s*_ = 0.147, *P*=0.0237) patients. The medians of anti-HBc in pathological grades of G1, G2, and G3 and stages of S1, S2, S3, and S4 in HBeAg-positive patients were all significantly lower than those in HBeAg-negative patients (all *P* < 0.005). The areas under receiver-operating characteristic curves (95% confidence interval) of anti-HBc for predicting pathological grades ≥G2 and ≥G3, and stages ≥S2 and =S4 in HBeAg-positive patients were 0.683 (0.622–0.740) and 0.662 (0.601–0.720), and 0.627 (0.564–0.687) and 0.683 (0.622–0.740), respectively, and in HBeAg-negative patients were 0.681 (0.618–0.740) and 0.702 (0.639–0.760), and 0.569 (0.503–0.633) and 0.630 (0.565–0.691), respectively.

**Conclusion:**

Following hepatic aggravation of necroinflammation and progression of fibrosis, anti-HBc increases gradually in HBeAg-positive patients and continues to increase gradually in HBeAg-negative patients, which is a useful but unsatisfactory marker for monitoring pathological states.

## 1. Background

Based on dynamic observation of hepatitis B e antigen (HBeAg) status, serum hepatitis B virus (HBV) DNA, and alanine transferase (ALT) levels, the natural history of chronic HBV infection can typically be divided into four successive phases: HBeAg-positive chronic infection, HBeAg-positive chronic hepatitis, HBeAg-negative chronic infection, and HBeAg-negative chronic hepatitis [1]. With reference to the similar criterion, the differences in some quantitative serum HBV markers such as hepatitis B surface antigen (HBsAg), hepatitis B core-related antigen (HBcrAg), hepatitis B core antigen antibody (anti-HBc), and HBV RNA during different phases of the natural history have been explored [2–10]. However, to date, most clinical practice guidelines on the management of chronic HBV infection with important international influence have not included these quantitative HBV markers as key parameters in the phase criteria of the natural history [11–14].

In fact, the disease evolvements during chronic HBV infection are often oscillatory. Whether HBeAg-positive or HBeAg-negative, the inactive chronic infection can occur with transient active chronic hepatitis; on the contrary, the active chronic hepatitis can occur with transient inactive chronic infection [1, 11–14]. Furthermore, influenced by the concept of “preconceptions,” the results of studies based on the criterion of the phases containing serum HBV DNA, due to potential relevance of HBV DNA with other HBV markers, cannot truly reflect the evolving patterns of other HBV markers during chronic HBV infection. Therefore, the rational investigation on dynamic changes of HBV markers including serum HBV DNA during chronic HBV infection should only be referring to liver pathological states and serum biochemical changes.

Recently, some studies have explored the predictive value of anti-HBc for liver pathological states in patients with chronic HBV infection from a practical perspective, but the performance of this has not been investigated in detail [15–20]. To further characterize the theoretical and practical value of anti-HBc, we comparatively depicted the evolving patterns of anti-HBc versus serum HBsAg and HBV DNA following liver inflammation intensities and fibrosis levels and evaluated the performance of anti-HBc versus serum HBsAg and HBV DNA in predicting liver inflammation intensities and fibrosis levels in patients with chronic HBV infection.

## 2. Patients and Methods

### 2.1. Study Population

A total of 577 Chinese patients with chronic HBV infection who underwent liver biopsy at Shanghai Public Health Clinical Center of Fudan University between January 2015 and December 2017 were retrospectively screened, among whom 86 patients with the following conditions were excluded: 23 with poor quality of biopsy specimens (biopsy length <10 mm), 8 with incomplete laboratory data, 5 coinfected with other forms of viral hepatitis (2 with hepatitis C, 1 with hepatitis D, and 2 with hepatitis E), 9 with nonalcoholic fatty liver disease (steatosis >5% of hepatocytes), 2 with significant alcohol consumption (>20 g per day), 6 with drug-induced liver injuries, and 33 receiving treatment with nucleosides/nucleotides, interferon alphas, and glycyrrhizinates in the last 6 months.

After exclusions, 491 patients were enrolled in this study. The diagnoses of all patients were in accordance with the standard elaborated in the EASL 2017 Clinical Practice Guidelines on the management of hepatitis B virus infection [1].

### 2.2. Pathological Diagnoses

Ultrasound-guided liver biopsy was performed using a one-second liver biopsy needle (16G). Biopsies collected were immediately transferred into plastic tubes and snap frozen. Biopsies were fixed in neutral formaldehyde, dehydrated in an ethanol gradient, made transparent with xylene, immersed in paraffin, sliced, and stained with hematoxylin and eosin and Masson trichrome. One experienced pathologist who was blinded to any clinical and laboratory information was assigned to interpret all biopsies. Liver pathological diagnoses referred to Scheuer standard [21], in which grade is used to depict the intensity of necroinflammation and stage is used to describe the level of fibrosis and architectural alteration; the grades include five levels from G0 to G4, and the stages include five levels from S0 to S4.

Intrahepatic HBsAg and hepatitis B core antigen (HBcAg) were detected by immunohistochemistry. Formalin-fixed paraffin-embedded sections were routinely dewaxed and rehydrated. After heat-induced antigen retrieval in sodium citrate (pH 6.0) buffer, sections were incubated with primary monoclonal antibody against HBsAg (clone1044/341, Novocastra) and rabbit polyclonal antibodies against HBcAg (Dako). After washing, the polymer detection system (Polink-1 HRP, GBI Labs) was incubated for 30 min at room temperature and developed with 3, 3′-diaminobenzidine (DAB). Expression of intrahepatic HBsAg and HBcAg was assessed by the semiquantitative scoring method. The scores include four levels: 0 (no positive cells), 1 (positive cells <25%), 2 (positive cells 25%–49%), and 3 (positive cells ≥50%).

### 2.3. Laboratory Assays

Serum samples used for measurements were taken within 1 day before and 1 day after liver biopsy and stored at −40°C. Anti-HBc was measured using chemiluminescence microparticle immunoassay (CMIA) in a UMIC Caris200 automated analyzer (United Medical Instruments Co., Ltd, Xiamen, China), and anti-HBc kits were kindly provided by Innodx Biotech Co. Ltd. (Xiamen, China); the detection range was 1.0 × 10^2^ to 1.0 × 10^5^ IU/mL. HBsAg and HBeAg were measured using CMIA in an Abbott Architect I2000 automated analyzer (Abbott Laboratories, Chicago, USA), and the reagents were purchased from Abbott Laboratories (Chicago, USA); the detection range of HBsAg was 0.05 to 250 IU/mL, and if the serum exceeded the upper limit of detection, it was diluted 500 times and retested; the lower detection limit of HBeAg was 1.0 S/CO. HBV DNA was measured using PCR-fluorescence probing assay in a Roche LightCycler480 qPCR system (Roche Diagnostics Ltd., Rotkreuz, Switzerland), and the HBV DNA kits were purchased from Sansure Biotech Inc. (Changsha, China); the detection range was 5.0 × 10^2^ to 2.0 × 10^9^ IU/mL.

Serum ALT, aspartate transferase (AST), gamma-glutamyl transpeptidase (GGT), and cholinesterase (ChE) of biochemical parameters were measured using a Hitachi 7600 automated biochemist analyzer (Hitachi, Tokyo, Japan); the normal ranges were 9 to 50 IU/L, 15 to 40 IU/L, 10 to 60 IU/L, and 4.000 to 15.000 kU/L, respectively. Blood platelet (PLT) was measured using a Sysmex-XT 4000i automated hematology analyzer (Mundelein, IL, USA); the normal range was 125 × 10^9^/L to 350 × 10^9^/L.

### 2.4. Statistical Analyses

Quantitative variables were expressed as median (interquartile range (IQR)), and categorical variables were expressed as proportions. Fisher's *Z* test was used to compare the differences in Spearman correlation coefficients between anti-HBc, serum HBsAg and HBV DNA, and intrahepatic HBsAg and HBcAg with pathological grades and stages. The Kruskal–Wallis test was used to compare the differences in medians of anti-HBc among different liver pathological grades and stages. The Mann–Whitney *U* test was used to compare the differences in medians of anti-HBc of the same pathological grades and stages between HBeAg-positive and HBeAg-negative patients. Receiver-operating characteristic (ROC) curve was used to evaluate the validities of anti-HBc, serum HBsAg, and HBV DNA in predicting liver necroinflammation intensities and fibrosis levels. The paired-samples Delong *Z* test was used to compare the differences in areas under ROC curves (AUCs) between anti-HBc, serum HBsAg, and HBV DNA in predicting the same hepatic necroinflammation intensities and fibrosis levels. Medcalc version 15.8 (MedCalc Software, Broekstraat, Mariakerke, Belgium) was used for statistical analyses and graphic productions. A two-sided *P* value of <0.05 was considered statistically significant.

## 3. Results

### 3.1. Clinical, Laboratory, and Pathological Characteristics of Study Population

The clinical, laboratory, and pathological data of the study population based on HBeAg status are summarized in Table 1.

The frequency of pathological grades ≥G2 and ≥G3 of HBeAg-positive patients (39.8% and 6.3%) was significant greater than and close to that of HBeAg-negative patients (23.2% and 6.8%) (*χ*^2^ = 14.750, *P*=0.0001, and *χ*^2^ = 0.000, *P*=0.9842), respectively; the frequency of pathological stages ≥S2 of HBeAg-positive patients (54.7%) was significantly greater than that of HBeAg-negative patients (40.9%) (*χ*^2^ = 8.804, *P*=0.0030), and the frequency of pathological stages ≥S3 and S4 of HBeAg-positive patients (19.7% and 13.4%) was both not statistically different from that of HBeAg-negative patients (21.1% and 14.3%) (*χ*^2^ = 0.076, *P*=0.782, and *χ*^2^ = 0.031, *P*=0.8595).

### 3.2. Correlation of Anti-HBc with Liver Pathological Grades and Stages

The Spearman correlation coefficients of anti-HBc, serum HBsAg and HBV DNA, and intrahepatic HBsAg and HBcAg with liver pathological grades and stages were summarized in Table 2. The differences in Spearman correlation coefficients among anti-HBc, serum HBsAg and HBV DNA, and intrahepatic HBsAg and HBcAg with liver pathological grades and stages were compared (Table 2).

### 3.3. Differences in Anti-HBc among Liver Pathological Grades and Stages

**Figure 1:**
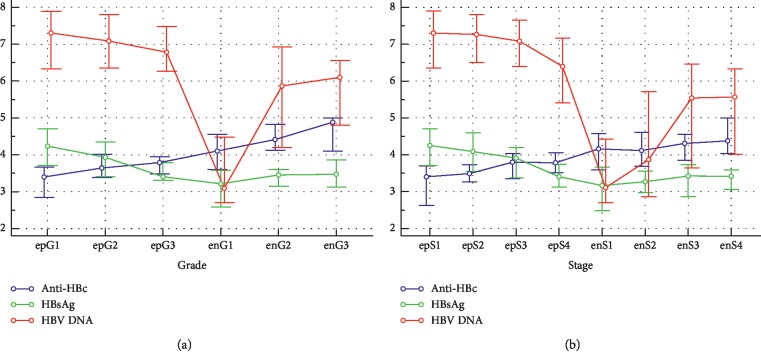
Multiple variables graph of anti-HBc and serum HBsAg and HBV DNA clustered by liver pathological grades (a) and stages (b) of HBeAg-positive and HBeAg-negative patients. The vertical axis represents anti-HBc and serum HBsAg and HBV DNA levels, the units of measurement of which are all log_10_ IU/mL; the small circles represent the medians, and the horizontal lines above and below the small circles represent the quartiles. Horizontal axis represents pathological grades and stages, where epG1, epG3, epG3 and epS1, epS2, epS3, epS4 represent G1, G3, G3 and S1, S2, S3, S4 of HBeAg-positive patients, respectively, and enG1, enG3, enG3 and enS1, enS2, enS3, enS4 represent G1, G3, G3 and S1, S2, S3, S4 of HBeAg-negative patients, respectively.

The differences in medians of anti-HBc among different liver pathological grades and stages were summarized in Table 3. The differences in medians of anti-HBc of the same pathological grades and stages between HBeAg-positive and HBeAg-negative patients were compared (Table 3). The evolving patterns of anti-HBc and serum HBsAg and HBV DNA following pathological grades and stages and HBeAg status were illustrated in Figure 1.

### 3.4. Performance of Anti-HBc in Predicting Liver Pathological States

**Figure 2:**
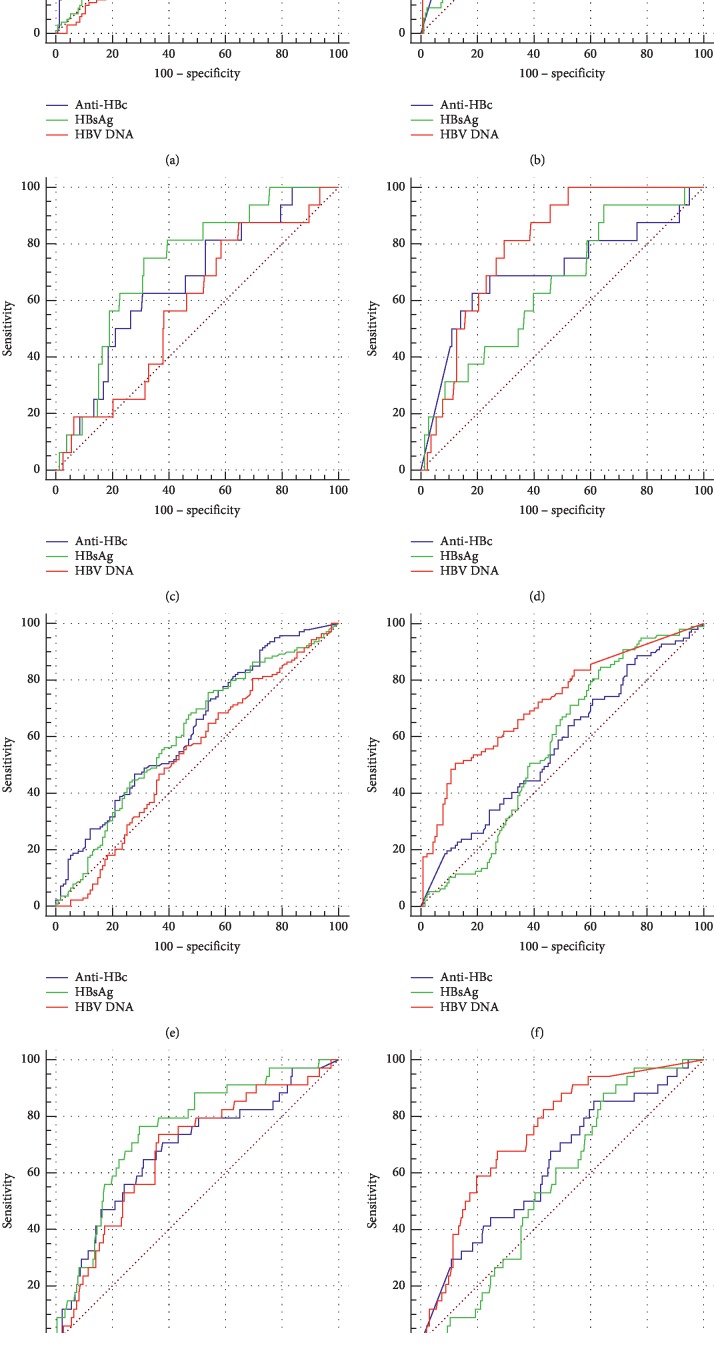
ROC curves of anti-HBc serum HBsAg and HBV DNA for predicting pathological grades ≥G2 (a, b), ≥G3 (c, d) and stages ≥S2 (e, f), =S4 (g, h) of HBeAg-positive (a, c, e, g) and HBeAg-negative (b, d, f, h) patients.

The ROC curves of anti-HBc, serum HBsAg, and HBV DNA for predicting liver pathological grades ≥G2 and ≥G3 and stages ≥S2 and =S4 were illustrated in Figure 2. AUCs of anti-HBc, serum HBsAg, and HBV DNA for predicting liver pathological grades ≥G2 and ≥G3 and stages ≥S2 and =S4 were summarized in Table 4. Of HBeAg-positive patients, the AUCs of anti-HBc and serum HBsAg for predicting liver pathological grades ≥G2 and ≥G3 and stages ≥S2 and =S4 and of serum HBV DNA for predicting pathological stages =S4 were all significantly greater than the area under diagonal reference line (all *P* < 0.05). Of HBeAg-negative patients, the AUCs of anti-HBc for predicting pathological grades ≥G2 and ≥G3 and stages =S4, and of serum HBsAg for predicting pathological grades ≥G2 and ≥G3, and of serum HBV DNA for predicting pathological grades ≥G2 and ≥G3 and stages ≥S2 and =S4 were all greater than the area under diagonal reference line (all *P* < 0.05) (Figure 2, Table 4).

**Figure 3:**
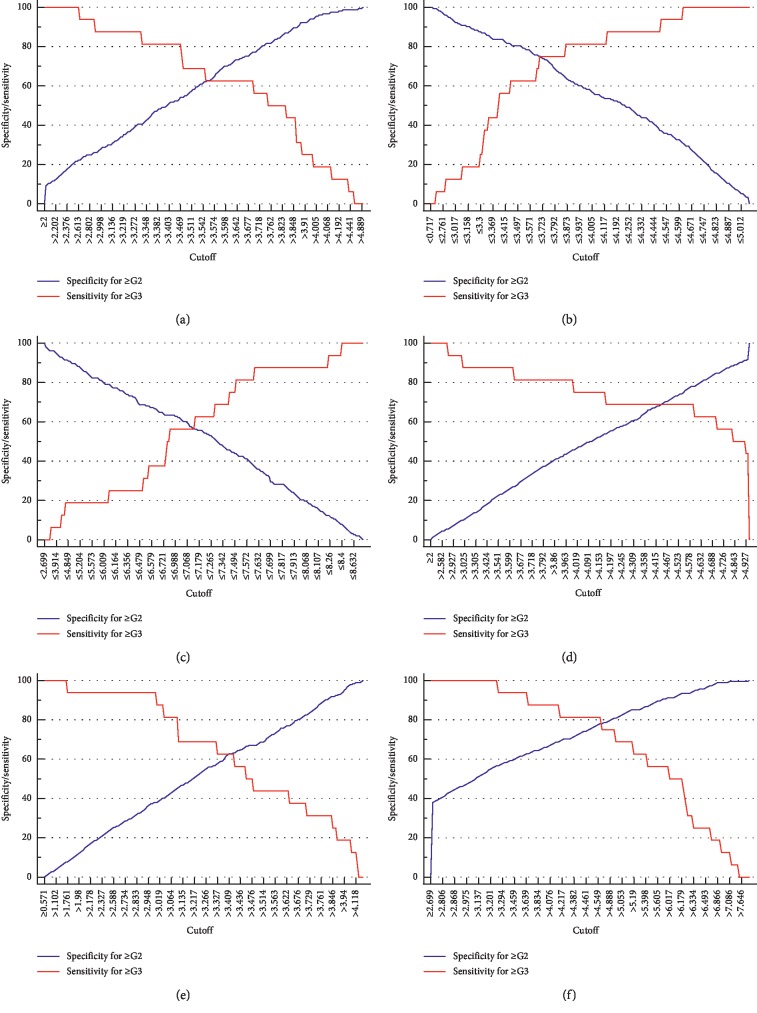
Scatter plot of specificities of predicting pathological grades ≥G2 and of sensitivities of predicting pathological grades ≥G3 corresponding to cutoffs of anti-HBc (a, b), serum HBsAg (c, d), and HBV DNA (e, f) in HBeAg-positive (a, c, e) and HBeAg-negative (b, d, f) patients.

With reference to the minimum differences in specificities of predicting pathological grades ≥G2 and sensitivities of predicting pathological grades ≥G3 or in specificities of predicting pathological stages ≥S2 and sensitivities of predicting pathological stages =S4 at the same cutoffs, a single tradeoff cutoff was determined (Figure 3) [22, 23]. The corresponding diagnostic parameters based on a single tradeoff cutoff in predicting pathological grades ≥G2 and ≥G3 or pathological stages ≥S2 and =S4 of HBeAg-positive and HBeAg-negative patients were calculated (Table 4).

Of HBeAg-positive patients, with reference to the tradeoff cutoffs, the sensitivity and specificity of serial test of combination of anti-HBc and serum HBsAg for predicting pathological grades ≥G2 were 28.2% and 90.8% and of parallel test of which for predicting pathological grades ≥G3 were 81.3% and 37.9%, respectively; the sensitivity and specificity of serial test of combination of anti-HBc and serum HBsAg for predicting pathological grades ≥S2 were 21.1% and 91.6% and of parallel test of combination for predicting pathological grades ≥S4 were 87.2% and 44.2%, respectively.

Of HBeAg-negative patients, with the standard of the tradeoff cutoffs, the sensitivity and specificity of serial test of combination of anti-HBc and serum HBV DNA for predicting pathological grades ≥G2 were 35.5% and 93.1% and of parallel test of combination for predicting pathological grades ≥G3 were 87.6% and 46.7%, respectively; the sensitivity and specificity of serial test of combination of anti-HBc and serum HBV DNA for predicting pathological grades ≥S2 were 28.8% and 87.4% and of parallel test of combination for predicting pathological grades ≥S4 were 76.7% and 37.5%, respectively.

## 4. Discussion

The host's immune response against HBV is the main cause leading to liver injury of HBV infection [24, 25]. The lowly efficient immune response against HBV in patients with chronic HBV infection, which lead not only to HBV persistence, but also to the disease oscillation, has not been clarified [24–26]. Some laboratory evidences have demonstrated that serum HBsAg that is overexpressed in chronic HBV infection and serum HBeAg that is not essential for HBV replication suppress host's immune responses against HBV [26–30]. Theoretically, anti-HBc levels may reflect the efficiency of host's immune responses against HBV to some extent.

Some studies have investigated the correlation of serum HBsAg and HBV DNA with liver pathological grades and stages in patients with chronic HBV infection [31–36]. The results of this study were basically consistent with those studies. However, few studies comparatively investigated the correlation of anti-HBc versus serum HBsAg and HBV DNA, and intrahepatic HBsAg and HBcAg with liver pathological grades and stages. This study showed that, in HBeAg-positive patients, anti-HBc was significantly positively correlated, but serum HBsAg and HBV DNA and intrahepatic HBcAg were all significantly negatively correlated with both pathological grades and stages; however, in HBeAg-negative patients, anti-HBc and serum HBV DNA and intrahepatic HBsAg and HBcAg were all significantly positively correlated with both pathological grades and stages, and serum HBsAg was significantly positively correlated pathological grades. The medians of anti-HBc of the same pathological grades and stages in HBeAg-positive patients were all significantly lower than those in HBeAg-negative patients.

The findings of this study demonstrated that the virological and immunological mechanisms of progression and oscillation of hepatic necroinflammation and fibrosis in HBeAg-positive patients could be different from those in HBeAg-negative patients. Serum HBsAg and HBeAg may play leading and synergistic role in immune regulation, respectively, while intrahepatic HBcAg may serve as the primary target antigen for immune responses [8, 9, 31, 32, 36]. It is postulated that, in HBeAg-positive patients, spontaneous decrease of serum HBsAg could evoke host's immune responses against HBV, which lead to decrease of serum HBsAg and HBeAg and of intrahepatic HBsAg and HBcAg, and decrease of HBV replication with hepatic aggravation of necroinflammation and progression of fibrosis, until serum HBsAg remain lower levels and HBeAg loss or convert. However, in HBeAg-negative patients, increase of intrahepatic HBcAg resulting from opportunistic increase of HBV replication could evoke again host's immune responses against HBV, which lead to decrease of serum HBsAg and of intrahepatic HBsAg and HBcAg, and decrease of HBV replication with hepatic reaggravation of necroinflammation and reprogression of fibrosis, until serum HBsAg remain again lower levels or HBsAg loss or convert.

Much progress has been made in the noninvasive assessment of hepatic fibrosis levels based on the disease-specific indicators and mathematical models [13, 14, 22, 23, 37, 38]. However, the study on serum HBV markers, especially anti-HBc, the only virological marker that can reflect the host's immune efficiency, for predicting hepatic necroinflammation intensities and fibrosis levels has special theoretical and practical importance for further elucidating and monitoring progression and oscillation during chronic HBV infection.

Previous studies have shown that serum HBsAg of HBeAg-positive patients and serum HBV DNA of HBeAg-negative patients are valuable but not very satisfactory in predicting liver inflammation intensities and fibrosis levels [31–36]. Lately, preliminary studies have demonstrated that anti-HBc may be useful in predicting liver inflammation intensities and fibrosis levels [15–20]. This study indicated that the AUC of anti-HBc of HBeAg-positive patients in predicting pathological grades ≥G2 and ≥G3 was both close to that of serum HBsAg and both significantly greater than that of serum HBV DNA, and in predicting pathological stages ≥S2 and =S4 was both close to that of serum HBsAg and, respectively, significantly greater than and close to that of serum HBV DNA. However, the AUC of anti-HBc of HBeAg-negative patients in predicting pathological grades ≥G2 and ≥G3 was both close to that of serum HBsAg and, respectively, significantly less than and close to that of serum HBV DNA, and in predicting pathological stages ≥S2 and =S4 was both close to that of serum HBsAg and both significantly less than that of serum HBV DNA. These data suggested that the validity of anti-HBc in predicting liver necroinflammation intensities and fibrosis levels of HBeAg-positive patients be close to that of serum HBsAg and superior to that of serum HBV DNA, and of HBeAg-negative patients be inferior to that of serum HBV DNA but not inferior to that of serum HBsAg.

Clear diagnosis of significant hepatic necroinflammation or fibrosis and timely diagnosis of extensive hepatic necroinflammation or cirrhosis is of great practical importance for the rational intervention of antiviral therapy [39, 40]. Therefore, this study selected single tradeoff cutoffs [22, 23], which minimize not only the misdiagnosis for predicting significant hepatic necroinflammation and fibrosis (pathological grades ≥G2 and stages ≥S2) but also the missed diagnosis for predicting extensive hepatic necroinflammation and cirrhosis (pathological grades ≥G3 and stages =S4), to evaluate the performance of anti-HBc and serum HBsAg and HBV DNA in predicting liver necroinflammation intensities and fibrosis levels.

In this study, with reference to the tradeoff cutoffs, the specificity of anti-HBc of HBeAg-positive patients for predicting significant liver necroinflammation and fibrosis and the sensitivity of which in predicting extensive liver necroinflammation and cirrhosis was all less than that of serum HBsAg and greater than that of serum HBV DNA; the specificity of anti-HBc of HBeAg-negative patients for predicting significant liver necroinflammation and fibrosis and the sensitivity of which in predicting extensive liver necroinflammation and cirrhosis was all no less than that of serum HBsAg and less than that of serum HBV DNA. It should be noted that the specificity of serum HBsAg of HBeAg-positive patients and serum HBV DNA of HBeAg-negative patients for predicting significant liver necroinflammation and fibrosis and the sensitivity of which in predicting extensive liver necroinflammation and cirrhosis was all less than 80%. However, the serial test of combination of anti-HBc and serum HBsAg of HBeAg-positive patients and of anti-HBc and serum HBV DNA of HBeAg-negative patients can all achieve satisfactory specificities for predicting significant liver necroinflammation and fibrosis, and the parallel test of combination of which can also obtain satisfactory sensitivity for predicting extensive liver necroinflammation and cirrhosis.

This study has some limitations. Firstly, synchronous investigation of other HBV markers such as serum HBcrAg and HBV RNA as well as intrahepatic HBV DNA, HBV RNA, and HBV cccDNA could be more useful for elucidating the role of anti-HBc in liver pathological grades and stages. Secondly, combined investigation with HBeAg-stopping mutant of G1896A in precore region or HBeAg-suppressing mutants of A1762T and G1764A in basal core promoter region of the HBV genome as well as HBV genotypes could be more helpful for explaining the role of anti-HBc in liver inflammation intensities and fibrosis levels. Thirdly, dynamic observation of the evolving patterns of anti-HBc versus other HBV markers could provide more valuable information for revealing the role of anti-HBc in the exacerbation and alleviation of liver necroinflammation as well as the progression and regression liver fibrosis.

## 5. Conclusions

This study revealed that, following aggravation of liver necroinflammation and progression of liver fibrosis, anti-HBc increases gradually in HBeAg-positive patients and further increases gradually in HBeAg-negative patients. Anti-HBc is a valuable but unsatisfactory indicator in predicting liver necroinflammatory intensities and fibrosis levels in both HBeAg-positive and HBeAg-negative patients; however, anti-HBc can improve the performance of serum HBsAg of HBeAg-positive patients and serum HBV DNA of HBeAg-negative patients in predicting liver necroinflammatory intensities and fibrosis levels.

## Figures and Tables

**Table 1:** Clinical, laboratory, and pathological characteristics of study population.

Variables	HBeAg-positive (*n* = 254)	HBeAg-negative (*n* = 237)	*χ* ^2a^ * /Z* ^b^	*P* ^#^
Gender (male : female)	163 : 91	145 : 92	0.350^a^	0.5540
Age (years), median (IQR)	33 (28–39)	42 (35–49)	9.475^b^	<0.0001
Serum ALT (IU/L), median (IQR)	67.5 (40.0–154.0)	35.0 (20.0–93.5)	6.587^b^	<0.0001
Serum AST (IU/L), median (IQR)	46.0 (29.0–89.0)	28.0 (19.0–56.3)	6.161^b^	<0.0001
Serum GGT (IU/L), median (IQR)	31.0 (18.0–71.0)	26.0 (16.8–55.3)	1.751^b^	0.0799
Serum ChE (kU/L), median (IQR)	7.525 (6.493–9.086)	8.378 (6.785–9.669)	3.177^b^	0.0015
Blood PLT (×10^9^/L), median (IQR)	171 (139–207)	169 (127–208)	1.017^b^	0.3091
Serum anti-HBc (log_10_ IU/L), median (IQR)	3.526 (3.137–3.806)	4.222 (3.668–4.641)	10.747^b^	<0.0001
Serum HBsAg (log_10_ IU/L), median (IQR)	4.010 (3.446–4.596)	3.282 (2.762–3.615)	11.902^b^	<0.0001
Serum HBV DNA (log_10_ IU/L), median (IQR)	7.228 (6.336–7.806)	3.565 (<2.699–5.235)	15.327^b^	<0.0001
Intrahepatic HBsAg (0 : 1 : 2 : 3)	3 : 54 : 95 : 102	17 : 104 : 82 : 34	60.061^a^	<0.0001
Intrahepatic HBcAg (0 : 1 : 2 : 3)	103 : 70 : 64 : 17	207 : 27 : 3 : 0	126.052^a^	<0.0001
Pathological grade (0 : 1 : 2 : 3 : 4)	0 : 153 : 85 : 16 : 0	0 : 182 : 39 : 16 : 0	19.009^a^	0.0001
Pathological stage (0 : 1 : 2 : 3 : 4)	0 : 115 : 89 : 16 : 34	0 : 140 : 47 : 16 : 34	14.851^a^	0.0019

IQR, interquartile range; ALT, alanine transferase; AST, aspartate transferase; GGT, gamma-glutamyl transpeptidase; ChE, cholinesterase; PLT, platelet; HBsAg, hepatitis B surface antigen; HBeAg, hepatitis B e antigen; HBcAg, hepatitis B core antigen; anti-HBc, antibodies to hepatitis B core antigen; HBV DNA, hepatitis B virus DNA. ^#^Comparison between HBeAg-positive and HBeAg-negative patients; ^a^Pearson chi-square test; ^b^Mann–Whitney *U* test.

**Table 2:** Differences in Spearman correlation coefficients among serum and intrahepatic HBV markers with pathological grades and stages.

Variable	HBeAg-positive (*n* = 254)				HBeAg-negative (*n* = 237)			
	Pathological grade		Pathological stage		Pathological grade		Pathological stage	
	*r*_*s*_	*P*	*r*_*s*_	*P*	*r*_*s*_	*P*	*r*_*s*_	*P*
Serum anti-HBc	0.312^ab^	<0.0001	0.268^c^	<0.0001	0.270^d^	<0.0001	0.147^e^	0.0237
Serum HBsAg	−0.248	0.0001	−0.272	<0.0001	0.183	0.0046	0.127	0.0503
Serum HBV DNA	−0.073^a^	0.2470	−0.128	0.0410	0.493^d^	<0.0001	0.411^e^	<0.0001
Intrahepatic HBsAg	0.017^b^	0.7825	0.030^c^	0.6380	0.212	0.0010	0.221	0.0006
Intrahepatic HBcAg	−0.270	<0.0001	−0.254	<0.0001	0.220	0.0007	0.169	0.0093

^a–e^Pairwise comparisons between different Spearman correlation coefficients, *P* < 0.05, by Fisher's *Z* test. a: *Z* = 2.797, *P*=0.0052; b: *Z* = 3.425, *P*=0.0006; c: *Z* = 2.741, *P*=0.0061; d: *Z* = 2.846, *P*=0.0044; e: *Z* = 3.123, *P*=0.0018.

**Table 3:** Differences in median of anti-HBc among different pathological grades and stages and between HBeAg-positive and HBeAg-negative patients.

	HBeAg-positive (*n* = 254)		HBeAg-negative (*n* = 237)		*Z*	*P* ^*∗*^
	*n*	Anti-HBc, median (IQR)	*n*	Anti-HBc, median (IQR)		
Grade						
G1	153	3.399 (2.849–3.669)^ab^	182	4.104 (3.598–4.557)^cd^	9.442	<0.0001
G2	85	3.643 (3.383–4.011)^a^	39	4.413 (4.125–4.829)^c^	6.213	<0.0001
G3	16	3.789 (3.478–3.948)^b^	16	4.885 (4.100–>5.000)^d^	3.138	0.0017
	*χ* ^2^	24.785	*χ* ^2^	17.305		
	*P* ^#^	<0.0001	*P* ^#^	0.0002		

Stage						
S1	115	3.403 (2.624–3.697)^ef^	140	4.160 (3.593–4.576)	8.184	<0.0001
S2	89	3.491 (3.265–3.731)^g^	47	4.116 (3.683–4.612)	5.226	<0.0001
S3	16	3.801 (3.347–4.039)^e^	16	4.308 (3.849–4.559)	2.864	0.0042
S4	34	3.785 (3.511–4.060)^fg^	34	4.378 (4.034–>5.000)	4.272	<0.0001
	*χ* ^2^	19.822	*χ* ^2^	6.886		
	*P* ^#^	0.0002	*P* ^#^	0.0756		

IQR, interquartile range. ^*#*^Comparison among different grades and stages, Kruskal–Wallis test. ^*∗*^Comparison between HBeAg-positive and HBeAg-negative patients, Mann–Whitney *U* test. ^a–g^Pairwise comparisons between different grades and stages, *P* < 0.05, by post hoc analysis.

**Table 4:** Performance of serum anti-HBc, HBsAg, and HBV DNA for predicting liver pathological states.

Predicting pathological states of HBeAg-positive										
State variable	Test variable	ROC curve analyses			Tradeoff cutoffs (log_10_ IU/mL) and corresponding parameters					
		AUC	SE	95% CI	Cutoff	Sen (%)	Spe (%)	Ppv (%)	Npv (%)	Acc
≥G2	Anti-HBc	0.683^a^	0.0341	0.622–0.740	>3.558	59.4	62.8	51.3	70.1	0.606
	HBsAg	0.635^b^	0.0353	0.572–0.694	≤3.709	47.5	75.2	55.8	68.5	0.615
	HBV DNA	0.538^ab^	0.0365	0.475–0.601	≤7.117	51.5	56.2	43.7	63.7	0.535
≥G3	Anti-HBc	0.662	0.0683	0.601–0.720	>3.558	62.5	55.0	8.5	95.6	0.424
	HBsAg	0.729^c^	0.0576	0.670–0.783	≤3.709	75.0	68.9	14.0	97.6	0.522
	HBV DNA	0.579^c^	0.0704	0.516–0.640	≤7.117	56.3	53.8	7.6	94.8	0.410
≥S2	Anti-HBc	0.627^d^	0.0351	0.564–0.687	>3.594	48.9	67.8	64.8	52.3	0.574
	HBsAg	0.609^e^	0.0359	0.546–0.670	≤3.732	43.2	73.9	66.7	51.8	0.567
	HBV DNA	0.535^de^	0.0370	0.472–0.597	≤6.782	41.7	64.4	58.6	47.7	0.518
=S4	Anti-HBc	0.683	0.0523	0.622–0.740	>3.594	67.7	62.7	21.9	92.6	0.539
	HBsAg	0.751	0.0435	0.693–0.803	≤3.732	73.5	70.5	27.8	94.5	0.605
	HBV DNA	0.675	0.0496	0.613–0.732	≤6.782	64.7	65.0	22.2	92.3	0.547

Predicting pathological states of HBeAg-negative										
State variable	Test variable	ROC curve analyses			Tradeoff cutoffs (log_10_ IU/mL) and corresponding parameters					
		AUC	SE	95% CI	Cutoff	Sen (%)	Spe (%)	Ppv (%)	Npv (%)	Acc

≥G2	Anti-HBc	0.681^f^	0.0410	0.618–0.740	>4.454	52.7	68.7	33.7	82.8	0.581
	HBsAg	0.622^g^	0.0401	0.557–0.684	>3.411	56.4	62.6	31.3	82.6	0.557
	HBV DNA	0.833^fg^	0.0287	0.780–0.878	>4.637	67.3	78.0	48.1	88.7	0.696
≥G3	Anti-HBc	0.702	0.0841	0.639–0.760	>4.454	68.8	66.1	12.8	96.7	0.500
	HBsAg	0.653^h^	0.0718	0.589–0.713	>3.411	62.5	59.7	10.1	95.7	0.455
	HBV DNA	0.800^h^	0.0421	0.743–0.849	>4.637	75.0	70.6	15.6	97.5	0.539
S2	Anti-HBc	0.569^i^	0.0378	0.503–0.633	>4.309	47.4	57.1	43.4	61.1	0.522
	HBsAg	0.572^j^	0.0371	0.506–0.635	>3.320	52.6	55.7	45.1	62.9	0.538
	HBV DNA	0.723^ij^	0.0338	0.661–0.779	>4.152	60.8	70.7	59.0	72.3	0.657
S4	Anti-HBc	0.630^k^	0.0521	0.565–0.691	>4.309	55.9	57.1	17.9	88.5	0.483
	HBsAg	0.566^l^	0.0441	0.500–0.630	>3.320	55.9	53.7	16.8	87.9	0.462
	HBV DNA	0.746^kl^	0.0421	0.685–0.800	>4.152	70.6	62.6	24.0	92.7	0.553

AUC, areas under ROC curve; SE, standard error; 95% CI, 95% confidence interval; Sen, sensitivity; Spe, specificity; Ppv, positive predictive value; Npv, negative predictive value; Acc, accuracy. ^a–l^Pairwise comparisons between different AUCs, *P* < 0.05, by the paired-samples Delong *Z* test. a: *Z* = 3.481, *P*=0.0005; b: *Z* = 3.245, *P*=0.0012; c: *Z* = 2.365, *P*=0.0180; d: *Z* = 2.238, *P*=0.0252; e: *Z* = 2.547, *P*=0.0109; f: *Z* = 3.676, *P*=0.0002; g: *Z* = 5.072, *P* < 0.0001; h: *Z* = 2.590, *P*=0.0096; i: *Z* = 4.266, *P* < 0.0001; j: *Z* = 3.491, *P*=0.0005; k: *Z* = 2.219, *P*=0.0265; l: *Z* = 3.431, *P*=0.0006.

## Data Availability

The data used to support the findings of this study are available from the corresponding author upon request.

## Abbreviations

ALT: Alanine transferase
Anti-HBc: Hepatitis B core antigen antibody
AST: Aspartate transferase
AUC: Areas under ROC curves
ChE: Cholinesterase
CI: Confidence interval
CMIA: Chemiluminescence microparticle immunoassay
GGT: Gamma-glutamyl transpeptidase
HBcAg: Hepatitis B core antigen
HBcrAg: Hepatitis B core-related antigen
HBeAg: Hepatitis B e antigen
HBsAg: Hepatitis B surface antigen
HBV: Serum hepatitis B virus
IQR: Interquartile range
PLT: Platelet
ROC: Receiver-operating characteristic
SE: Standard error.
